# Eukaryotic Translation Initiation Factor 3 Subunit E Controls Intracellular Calcium Homeostasis by Regulation of Cav1.2 Surface Expression

**DOI:** 10.1371/journal.pone.0064462

**Published:** 2013-05-30

**Authors:** Pawel Buda, Thomas Reinbothe, Vini Nagaraj, Taman Mahdi, Cheng Luan, Yunzhao Tang, Annika S. Axelsson, Daiqing Li, Anders H. Rosengren, Erik Renström, Enming Zhang

**Affiliations:** 1 Lund University Diabetes Center, Malmö, Sweden; 2 Key Lab of Hormones and Development, Ministry of Health, and Metabolic Diseases Hospital, Tianjin Medical University, Tianjin, China; Tohoku University, Japan

## Abstract

Inappropriate surface expression of voltage-gated Ca^2+^channels (Ca_V_) in pancreatic ß-cells may contribute to the development of type 2 diabetes. First, failure to increase intracellular Ca^2+^ concentrations at the sites of exocytosis impedes insulin release. Furthermore, excessive Ca^2+^ influx may trigger cytotoxic effects. The regulation of surface expression of Ca_V_ channels in the pancreatic β-cells remains unknown. Here, we used real-time 3D confocal and TIRFM imaging, immunocytochemistry, cellular fractionation, immunoprecipitation and electrophysiology to study trafficking of L-type Ca_V_1.2 channels upon β-cell stimulation. We found decreased surface expression of Ca_V_1.2 and a corresponding reduction in L-type whole-cell Ca^2+^ currents in insulin-secreting INS-1 832/13 cells upon protracted (15–30 min) stimulation. This internalization occurs by clathrin-dependent endocytosis and could be prevented by microtubule or dynamin inhibitors. eIF3e (Eukaryotic translation initiation factor 3 subunit E) is part of the protein translation initiation complex, but its effect on translation are modest and effects in ion channel trafficking have been suggested. The factor interacted with Ca_V_1.2 and regulated Ca_V_1.2 traffic bidirectionally. eIF3e silencing impaired Ca_V_1.2 internalization, which resulted in an increased intracellular Ca^2+^ load upon stimulation. These findings provide a mechanism for regulation of L-type Ca_V_ channel surface expression with consequences for β-cell calcium homeostasis, which will affect pancreatic β-cell function and insulin production.

## Introduction

Voltage gated calcium channels (Ca_V_) play a critical role in glucose-stimulated insulin secretion in pancreatic β-cells by activating Ca^2+^ influx upon membrane depolarization [1], [2]. Ca^2+^ influx is important for activating several physiological events such as pancreatic islet development and phasic insulin secretion [3], [4]. However, intracellular Ca^2+^ overload has detrimental effects and causes endoplasmic reticulum (ER) stress and initiates cytotoxicity [5], [6]. Dynamic Ca_V_ channel expression in the plasma membrane could be an effective way to regulate intracellular Ca^2+^ homeostasis and prevent adverse effects in the β-cell. Regulation of Ca_V_ channel surface expression is a more dynamic process than previously assumed and can also be of importance for short-term variations in Ca_V_ channel activity [7]. This could be of relevance for the respective phases of glucose-evoked secretion that in mouse are controlled by different Ca_V_ isoforms [4], [8]. For example, genetic ablation of Ca_V_1.2, one of the L-type Ca_V_ channels, strongly reduces first phase insulin release [8], [9]. Human ß -cells have an L-type calcium current component and mRNA for both L-type Ca_V_1.3 and Ca_V_1.2 can be detected in human islets [10]. Ca_V_1.2 denotes the Ca_V_ subunit isoform α_1C_, which determines the main electrophysiological and pharmacological properties of the channel and forms a heteromeric channel complex with the auxiliary subunits β, α2δ and γ [1]. Both β and α2δ subunits have been implicated in Ca_V_ channel transport to the plasma membrane [11], [12], [13].

eIF3e (Eukaryotic translation initiation factor 3 subunit E) is a subunit of the protein translation initiator complex that participates in the disassembly and recycling of posttermination ribosomal complexes and proteasome-mediated protein degradation [14], [15], [16]. eIF3e contains a highly conserved PCI domain, which binds the proteasome COP9 signaling complex that plays a central role in regulating ubiquitination and activation of proteolysis [17]. However, eIF3e has also been implicated in regulation of other cellular functions. For example, in neurons, eIF3e has been showed to influence Ca_V_1.2 expression in the synaptic membrane [18]. In adipocyte and vascular smooth muscle cells, the eIF3 complex can interact directly with mTOR, a critical signal molecule in controlling intracellular trafficking of glucose transporters [19], [20]. Whether eIF3e can affect Ca_V_1.2 translocation to/from the β-cell membrane and regulate β-cell physiology is not known. To address this possibility, we investigated the trafficking of Ca_V_1.2 in β-cells by a plethora of imaging and other methods and found that eIF3e is involved in depolarization-induced internalization of Ca_V_1.2, with consequences for β-cell intracellular Ca^2+^ homeostasis.

## Results

### Ca_V_1.2 Channel Clusters Internalize upon Glucose Stimulation in Insulin-secreting INS-1 832/13 Cells

**Figure 1:**
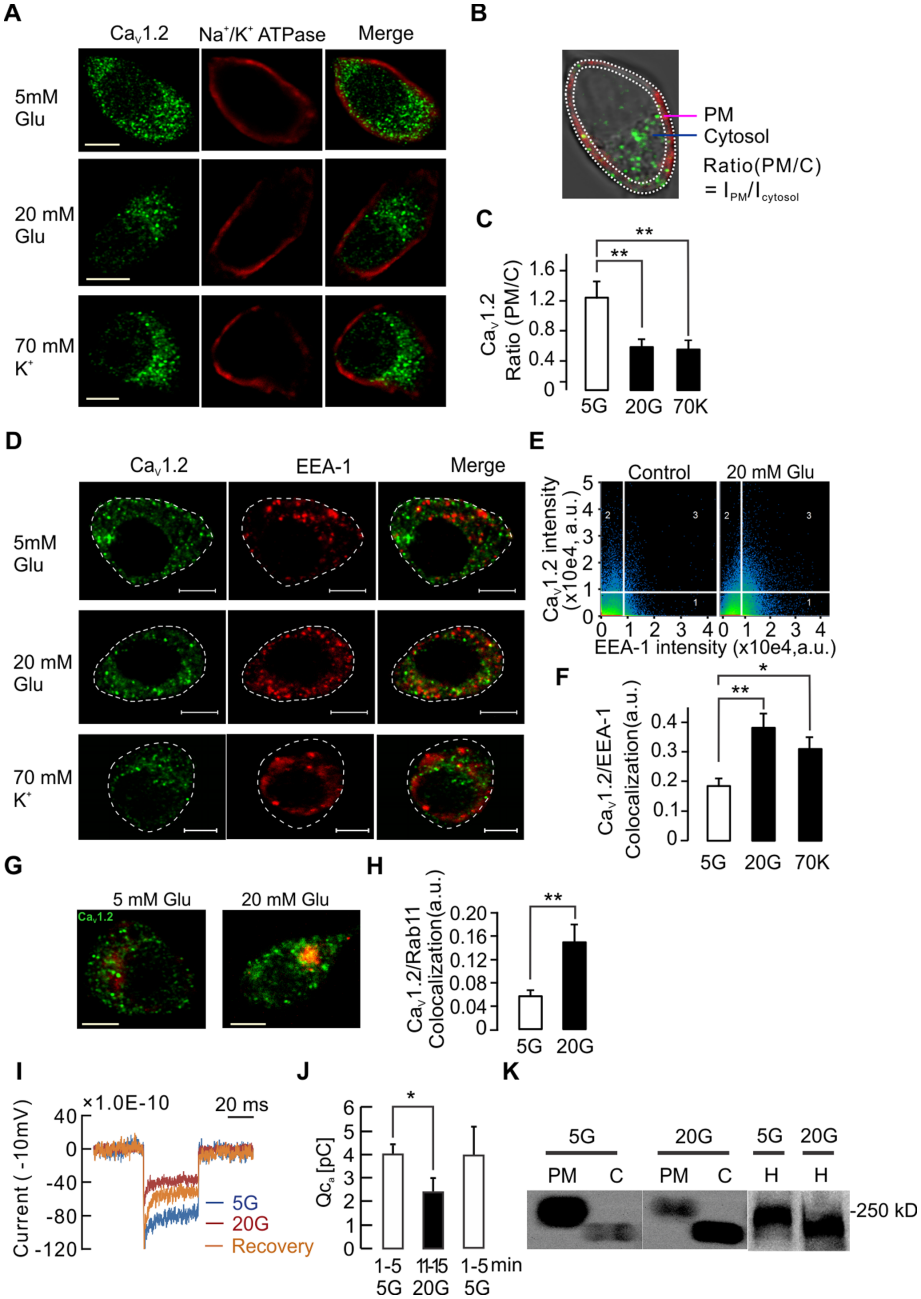
Protracted β-cell depolarization reduces surface expression of Ca_V_1.2 and voltage-gated Ca^2+^ currents. A) Immunostaining of Ca_V_1.2 and plasma membrane (PM) marker Na^+^/K^+^ ATPase in insulin-secreting INS-1 832/13 cells, before and after 30 min exposure to either 20 mM glucose or 70 mM KCl. B) Depicts the calculation of the ratio between the mean intensity of plasma membrane (I_PM_) to that of the cytosol (I_cytosol_). The PM area was defined by Na^+^/K^+^ ATPase staining. C) Average ratios of Ca_V_1.2 expression under the conditions in A, n = 12. ** p<0.01 (ANOVA, F-test). D) Immunostaining of Ca_V_1.2 and the early endosome marker EEA-1 before and after stimulation with 70 mM K^+^ or 20 mM glucose. E) Colocalization evaluated by Pearson’s correlation coefficient analysis which demonstrated colocalization of Ca_V_1.2 and EEA-1 in the area “3″ whereas “1″ or “2″ represent no colocalization. F) Average colocalization of Ca_V_1.2 to EEA-1 calculated under conditions as in (d), n = 10. * p<0.05, ** p<0.01 (ANOVA, F-test). G) Immunostaining of Ca_V_1.2 and the recycling endosome marker Rab11 before and after 20 mM glucose stimulation. H) Average Ca_V_1.2 colocalization to Rab11 detected under conditions as in G, n = 9. ** p<0.01. I) Representative Ca^2+^ currents measured by patch clamp under glucose stimulation. J) Average integrated whole-cell Ca^2+^ current (Q) in INS-1 832/13 cells before (n = 5 cells), during (n = 4 cells) and after (n = 4 cells) elevation of extracellular glucose from 5 to 20 mM. K) Immunoblotting of Ca_V_1.2 in plasma membrane (PM), cytosol (C) and homogenate (H) fractions before and after 20 mM glucose stimulation. Scale bar in all images 5 µm.

To quantify the number of Ca_V_1.2 clusters in the plasma membrane (PM) we first performed co-immunostaining of Ca_V_1.2 and the PM marker Na^+^/K^+^ ATPase (Fig. 1A–C). Then we analyzed the ratio of Ca_V_1.2 mean intensity in the PM over that in the cytosol to quantify internalization of Ca_V_1.2 in single INS-1 832/13 cells. This ratio was significantly decreased upon stimulation by 20 mM glucose or 70 mM KCl (from 1.26±0.22 to 0.58±0.08 or 0.51±0.1, respectively; n = 12 in each group). The decreases in Ca_V_1.2 surface expression were further confirmed by total internal reflection fluorescence microscopy (TIRFM) imaging (Figure S1). This internalization was also observed in immunostaining experiments for Ca_V_1.2 and the early endosome marker EEA-1 (Fig. 1D). In these experiments, colocalization of Ca_V_1.2 and EEA-1 increased upon stimulation with either glucose or high K^+^ (from 18.42±3.21 to 38±4.85 or 31.63±3.01; n = 10 in each group; Fig. 1E and F). These results suggest that the decreases in Ca_V_1.2 surface expression could be caused by endocytoic uptake. Indeed, we also observed increases in the colocalization of Ca_V_1.2 with the recycling endosome marker Rab11 (from 5.94±0.91 to 15.3±3.09; n = 9 in each group; Fig. 1G and H). We next measured voltage-gated Ca^2+^ influx using the standard whole-cell configuration of the patch clamp technique. These experiments demonstrated that the integrated Ca^2+^ current (Q_Ca2+_) elicited by voltage-clamp depolarizations decreased after 15-min exposure to high glucose (20 mM) (Fig. 1I and J), and was recovered after restoring glucose levels to basal. This finding supports the possibility that alterations in surface expression of L-type Ca_V_ channels may occur in response to glucose during protracted stimulation. This agrees with the data concerning Ca_V_1.2 in Figure 1A–H, but the contribution of other L-type channels, e.g. Ca_V_1.3 that is known to be expressed in beta-cells, can not be excluded. Evidence for activity-dependent trafficking of Ca_V_1.2 was also obtained by immunoblotting in subcellular fractions. The Ca_V_1.2 band detected in the PM is ∼20 kD heavier than that detected in the cytosol. This has been reported previously, but at present we do not have a full explanation for this finding. Nevertheless, this experiment showed that Ca_V_1.2 expression in the plasma membrane decreased when the cells were kept in 20 mM glucose for 30 min prior to fractionation, opposite to the intracellular expression of Ca_V_1.2 expression that increased by the same treatment (Fig. 1K).

**Figure 2:**
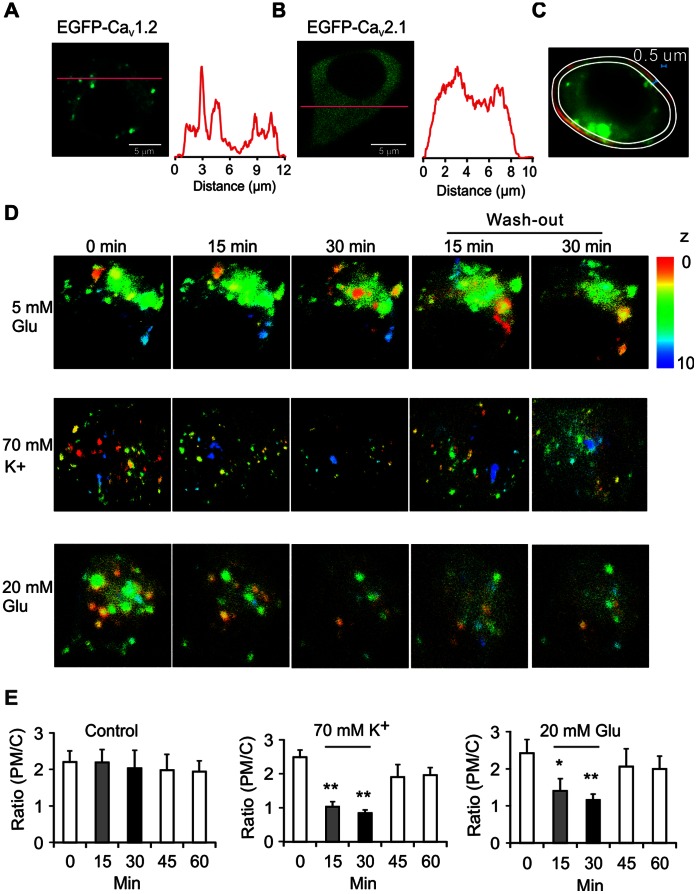
Visualization of Ca_V_1.2 cluster dynamic internalization in Ca_V_1.2 overexpressed β-cell. A) Transfected EGFP-Ca_V_1.2 expressed in cluster pattern in INS-1 832/13 cells. Intensity profile (right) along the red line in the image (left). B) Same as A, but the transfected plasmid is EGFP-Ca_V_2.1. C) Defining the plasma membrane region in living cells. Expression of Ca_V_1.2 in PM was evaluated by co-staining with a plasma membrane marker dye, CellMask® (Invitrogen). The region between the two white lines (0.5 µm distance) was defined as the surface region used for further analysis. D) Live 3D imaging of Ca_V_1.2 cluster expression in the cells. The pseudocolored clusters in the upper (red), middle (green) and bottom (blue) parts of the cells are shown in unstimulated control cells or 0, 15 and 30 min after stimulation with 20 mM glucose or 70 mM KCl respectively. The 30-min recovery experiments were performed by wash-out of the stimulation buffer. The color bar indicates real distance along the z-axis. E) The histograms show the average ratio PM/C in control cells (n = 16), or cells exposed to 70 mM KCl (n = 16) or 20 mM glucose (n = 12). * p<0.05, ** p<0.01 (ANOVA, F-test).

To observe dynamic internalization of Ca_V_1.2 in single cells, we employed three-dimensional (3D) live imaging in EGFP-Ca_V_1.2 transfected cells. This approach allows tracking of Ca_V_1.2 distribution in the entire cells, thereby avoiding confounding effects by Ca_V_1.2 transport out of the focal plane or by changes in cell shape during the experiment. First, Ca_V_1.2 clusters were observed 36 h after transfection (Fig. 2A). Interestingly, similarly EGFP-tagged P/Q-type Ca_V_2.1 channels did not form such clusters, but were diffusely distributed (Fig. 2B). Next, real-time 3D imaging of EGFP-Ca_V_1.2 was performed before and during stimulation with either high K^+^ (70 mM) or high glucose (20 mM), as well as after wash-out. The plasma membrane location of EGFP-Ca_V_1.2 location was determined using the marker CellMask (Fig. 2C). In untreated cells kept at basal (5 mM) glucose, the ratio of EGFP-Ca_V_1.2 fluorescence in the PM over that in the cell interior (Ratio PM/C) remained largely unchanged during the entire experiment (Fig. 2D and E, upper panels). By contrast, when the cells were treated with high K^+^, the Ca_V_1.2 clusters internalized and the PM/C ratio decreased by ∼60% after 15 and 30 min (Fig. 2D and E, as indicated). Similarly, stimulation with high glucose (20 mM) reduced the PM/C ratio of EGFP-Ca_V_1.2 fluorescence by ∼50% after 15 and 30 min (Fig. 2D and E, as indicated). Interestingly, after wash-out, surface expression of Ca_V_1.2 was partially recovered (Fig. 2D and E), which suggests that this is a reversible physiological reaction. Moreover, the same results were observed by TIRFM imaging (Figure S2).

### Ca_V_1.2 Cluster Internalization can be Inhibited by Blocking the Endocytotic Pathway

The data presented so far demonstrate that internalization of Ca_V_1.2 clusters in the plasma membrane occurs upon stimulation. Specifically, the results in Figure 1D and 1G suggest that Ca_V_1.2 is translocated to early and recycling endosomes. We next performed experiments to address the signals and mechanisms involved in this endocytotic process.

**Figure 3:**
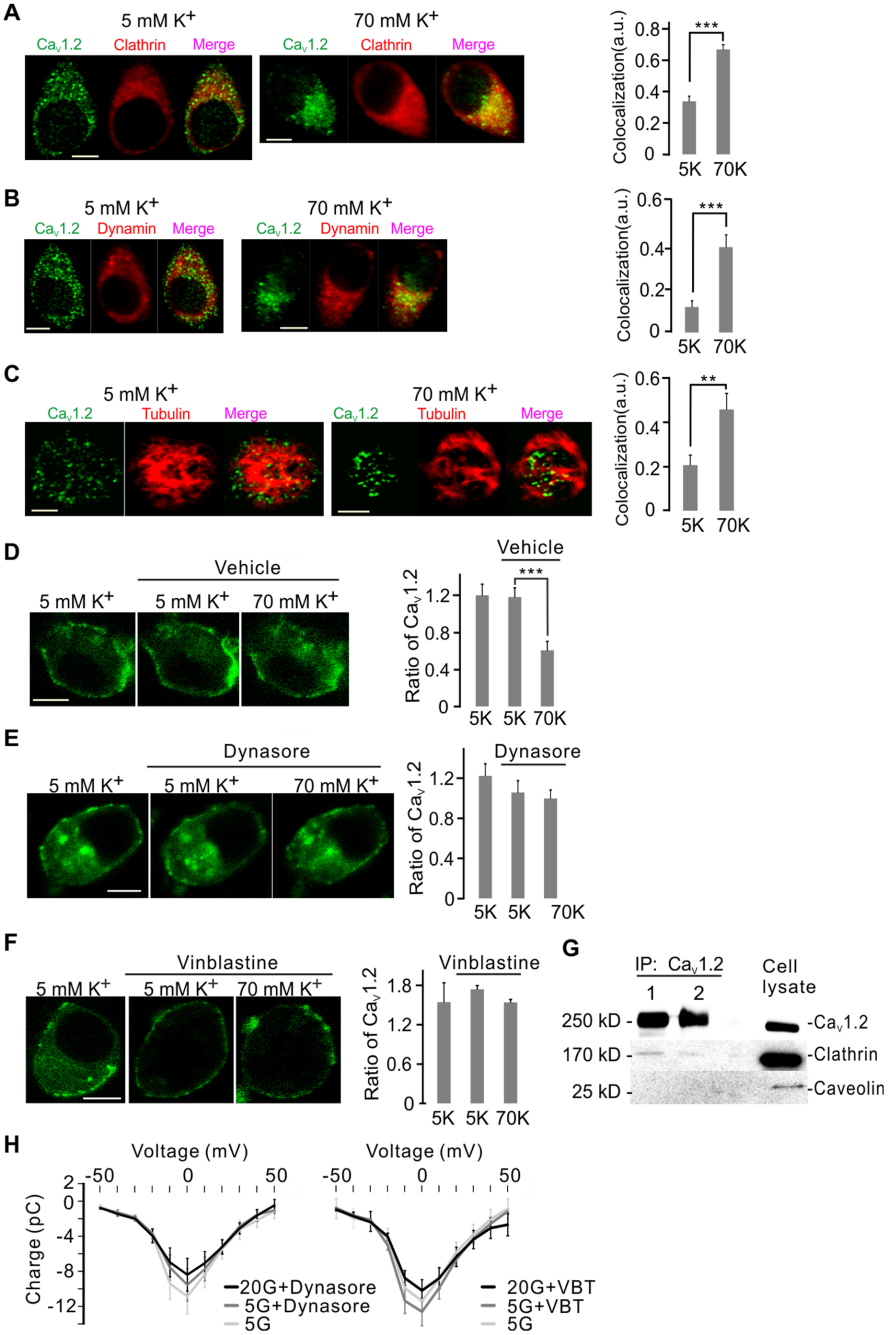
Depolarization-induced Ca_V_1.2 trafficking involves clathrin-dependent endocytosis. A) Immunostaining of Ca_V_1.2 and Clathrin in INS-1 832/13 cells upon 70 mM K^+^ stimulation. Colocalization (right) of Ca_V_1.2 to clathrin was measured by Pearson’s correlation coefficient analysis. n = 9, ***p<0.001. B) As in A, but colocalization was evaluated for Ca_V_1.2 to dynamin. n = 9, *** p<0.001. C) As in A, but colocalization was evaluated for Ca_V_1.2 to tubulin. n = 9, ** p<0.01. D) Distribution of EGFP-Ca_V_1.2 in a β-cell upon 70 mM K^+^ stimulation after 30-min pre-incubation with vehicle, DMSO. Ratios of PM/C (right) were calculated from 12 overexpressing cells, *** p<0.001 (ANOVA, F-test). E) As in D, but EGFP-Ca_V_1.2 and average ratios of PM/C (right) were evaluated after 30-min pre-incubation with the dynamin inhibitor dynasore (80 µM). n = 12, n.s. (ANOVA, F-test). F) As in D, but distribution of EGFP-Ca_V_1.2 (left) and average ratios of PM/C (right) were evaluated after pre-incubation with the microtubule assembly blocker vinblastine (VBT; 1 µM; 1 h). n = 12, n.s. (ANOVA, F-test). G) Immunoprecipitation of Ca_V_1.2 with clathrin and caveolin in INS-1 832/13 cells. Note that only clathrin but not caveolin co-immunoprecipitates with Ca_V_1.2 sorted proteins. 1 and 2 represent two independent experiments. H) Failure of long-term glucose treatment (20 mM for 30 min) to affect current-voltage (I-V) relations for whole-cell Ca^2+^ currents when measured after 30-min pre-incubation with the dynamin inhibitor dynasore (left) or after 1-h pre-incubation with the tubulin inhibitor vinblastin (VBT). Each trace represents the means±S.E.M. of at least 7 cells. Scale bar in all images 5 µm.

Endocytosis occurs by either the clathrin-dependent or caveolin-dependent pathways [21]. First, caveolin could not be detected (Fig. 3G). Next, we performed immunostaining of Ca_V_1.2 and clathrin in the presence of either 5 mM or 70 mM KCl (Fig. 3A). By contrast, clathrin was highly expressed and 33.7±3.26% of Ca_V_1.2 colocalized with clathrin (n = 9). Interestingly, the colocalization increased to 69.9±2.9% after stimulation with high K^+^ (n = 9). Furthermore, dynamin that cuts off clathrin-coated endocytotic pits [22], also increased its colocalization with Ca_V_1.2 after stimulation with high K^+^ (from 12.5±3.0% to 41.7±5.9%; n = 9 in each group; Fig. 3B). Ca_V_1.2 also revealed increased colocalization with the intracellular cytoskeletal component tubulin after stimulation, which increased from 20.6±4.5% to 45.7±7.2% (n = 9 in both groups; Fig. 3C). Next, we disrupted the endocytotic process using pharmacological inhibitors and then monitored the dynamic Ca_V_1.2 distribution in living cells. Interestingly, the dynamin inhibitor dynasore specifically blocked the stimulation-dependent Ca_V_1.2 internalization (Fig. 3E), as compared to control (Fig. 3D), and the microtubule synthesis inhibitor vinblastine (1 µM) [23] had the same effect (Fig. 3F). Moreover, immunoprecipitation experiments confirmed that Ca_V_1.2 interacts with clathrin rather than caveolin, which supports the involvement of clathrin-dependent endocytosis in Ca_V_1.2 internalization (Fig. 3G). Finally, in Figure 3H whole-cell Ca^2+^ current I-V relations demonstrated the failure of long-term glucose treatment to reduce the currents after treatment with dynasore or vinblastine. Taken together with the results in Figure 1 and 2, these results support the view that decreased surface expression of Ca_V_1.2, and perhaps other Ca_V_ channels, occurs by dynamin and microtubule-dependent endocytic uptake.

### Involvement of eIF3e in Glucose-evoked Ca_V_1.2 Internalization

**Figure 4:**
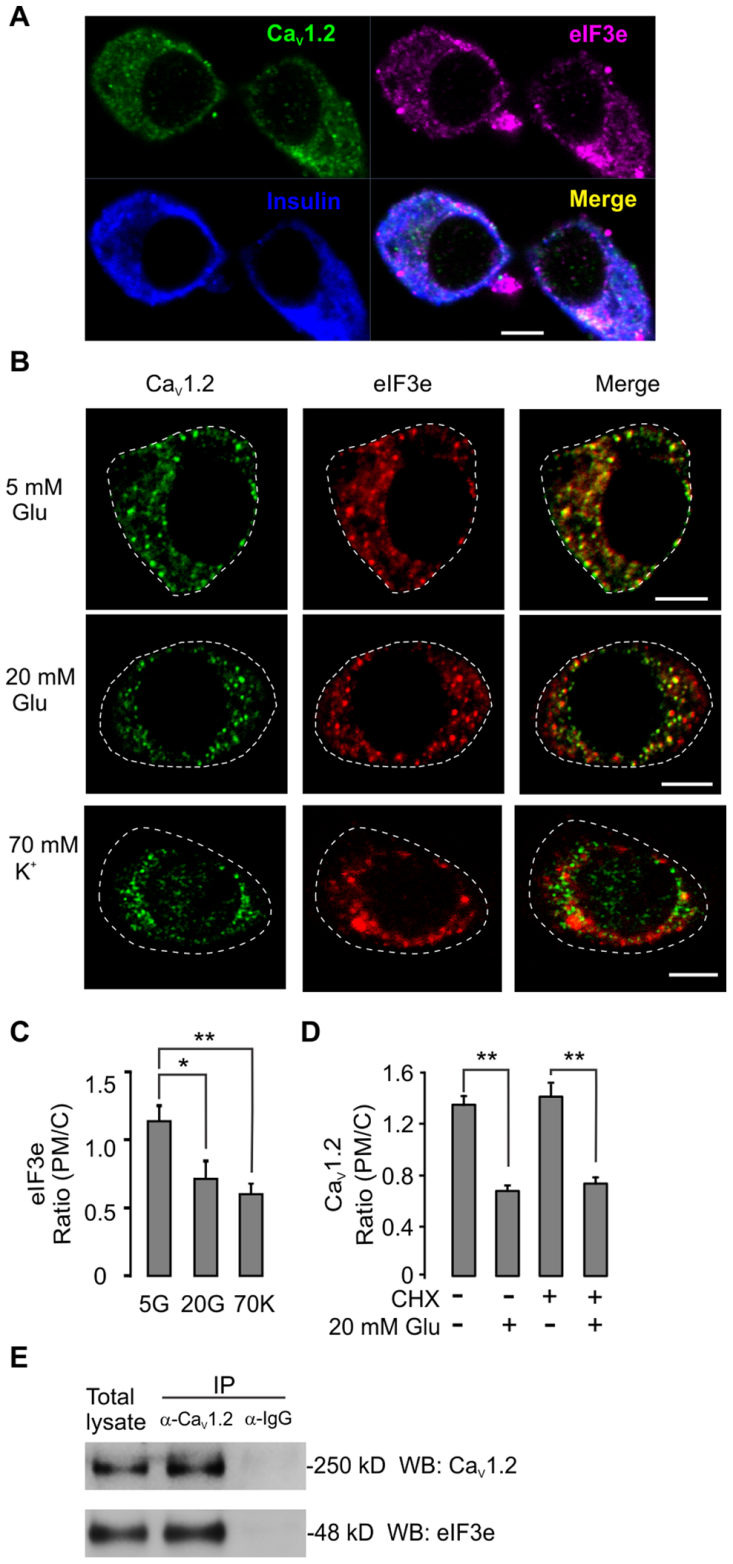
Eukaryotic translation initiation factor 3 subunit E (eIF3e) is expressed in INS-1 832/13 cells and interacts with Ca_V_1.2. A) Representative immunostaining of eIF3e in rat pancreatic beta cells. The rat pancreatic islet cells were isolated and seeded on glass cover slips overnight, followed by the standard staining protocol as described in methods. B) Co-immunostaining of Ca_V_1.2 and eIF3e in INS-1 832/13 cells after 30-min stimulation with 20 mM glucose or 70 mM K^+^. C) Average (PM/C) ratios of eIF3e localization after incubation at 20 mM glucose or 70 mM K^+^. n = 12. * p<0.05, ** p<0.01 (ANOVA, F-test). D) Average (PM/C) ratios under conditions as in B, but comparing cells treated with (+) or without (-) the protein translation inhibitor cyclohexamide (CHX; 60 µM; 1 h), n = 12. ** p<0.01 (ANOVA, F-test). E) Representative co-immunoprecipitation of Ca_V_1.2 and eIF3e in plasma membrane fractions under the resting conditions from 3 independent experiments.

Previous studies on activity-dependent Ca_V_ channel trafficking in neurons using yeast two-hybrid screening suggested eIF3e as a regulatory molecule in this process [18]. We therefore investigated the possible involvement of eIF3e in glucose-evoked Ca_V_1.2 internalization in the insulin-secreting cells. To this end, we first demonstrated high expression of eIF3e in primary rat pancreatic beta cells and INS-1 cells (Fig. 4A and B). Interestingly, the eIF3e distribution was also stimulation-dependent and the eIF3e ratio of PM/C expression dropped in response to stimulation with glucose or high K^+^ (from 1.17±0.11 to 0.69±0.14 or 0.6±0.07, respectively; n = 12 in each group; Fig. 4C). Furthermore this reduction of eIF3e expression on the cell surface was detected by TIRFM images (Figure S3). Previous reports demonstrate that eIF3e is not crucial for global protein synthesis [15], [24], but a role in protein translation cannot be excluded. To rule out that such an effect could explain our results we next used cycloheximide (CHX) to see whether general suppression of mRNA translation also interferes with Ca_V_1.2 trafficking. However, the PM/C ratio was unchanged compared to untreated cells both under basal and stimulated conditions. Furthermore, 20 mM glucose treatment effectively internalized Ca_V_1.2 also in CHX-treated cells (Fig. 4D). This result supports the view that the regulation of cellular Ca_V_1.2 distribution by eIF3e is based on the direct interaction with Ca_V_1.2 rather than by affecting Ca_V_1.2 mRNA translation. This view was reinforced by the fact that the two proteins partially colocalized (Fig. 4B). Direct evidence of their interaction was provided by co-immunoprecipitation experiments in which antibodies to Ca_V_1.2 specifically precipitated eIF3e (Fig. 4E).

**Figure 5:**
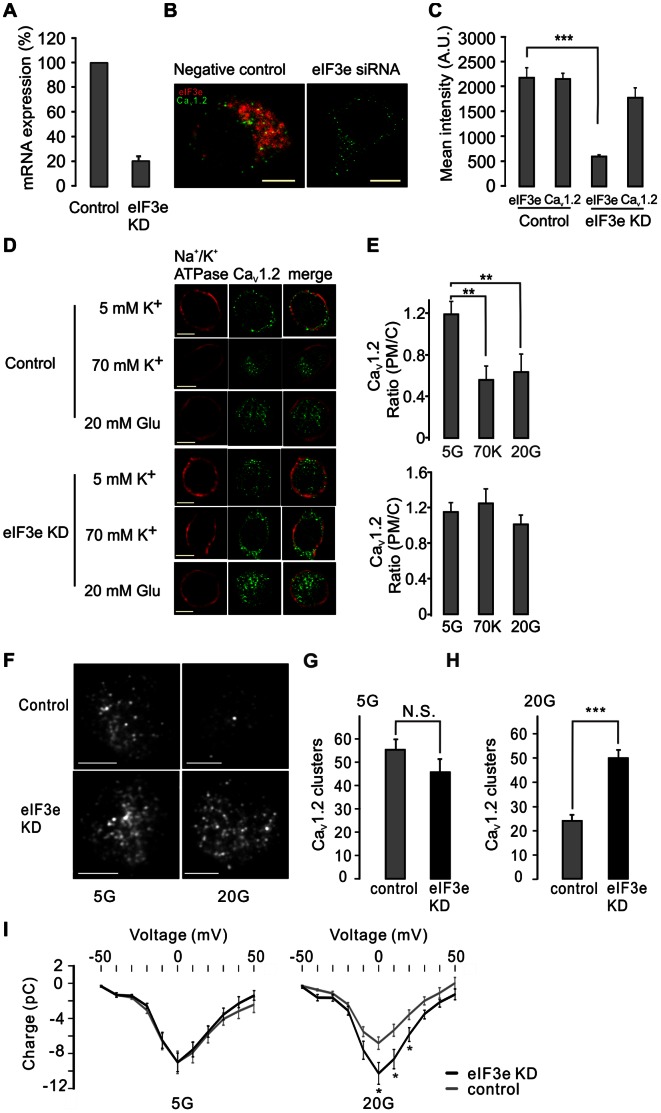
Knock down of eIF3e inhibits glucose-evoked Ca_V_1.2 cluster internalization. A) Knock-down of eIF3e by RNAi decreases mRNA level expression of eIF3e (eIF3e KD). Data from 3 independent experiments. B) Decreased eIF3e expression detected by immunostaining under conditions with or without eIF3e KD. C) Comparison of mean intensity of Ca_V_1.2 and eIF3e in the negative control and eIF3e KD cells. n = 18. ***, P<0.001 (ANOVA, F-test). D) Immunostaining of Ca_V_1.2 in control and eIF3e knock-down cells before and after 30-min stimulation with 20 mM glucose or 70 mM K^+^. Na^+^/K^+^ ATPase was used as a PM marker. E) Average (PM/C) ratios of Ca_V_1.2 under the conditions as in (d). n = 10. **, P<0.01 (ANOVA, F-test). F) Representative TIRFM image of Ca_V_1.2 in control and eIF3e KD cells under conditions with or without 30-min stimulation with 20 mM glucose. Quantitative analysis of Ca_V_1.2 clusters in control or eIF3e KD cells in the presence of 5 mM glucose (G) or 20 mM glucose (H) for 30 min prior to the experiment. Data indicate the number of Ca_V_1.2 clusters per cell and are presented as averages±S.E.M. *** p<0.001 (Student’s t-test). (I) Failure of long-term glucose treatment (20 mM for 30 min) to affect current-voltage (I-V) relations for whole-cell Ca^2+^ currents in eIF3e KD cells (black) compared to control (gray). Each trace represents the averages±S.E.M. of at least 7 cells. Scale bar in all images 5 µm.

To further address the role of eIF3e in Ca_V_1.2 internalization, we manipulated eIF3e expression by RNA interference in INS-1 832/13 cells. siRNAs against eIF3e silenced eIF3e gene expression (mRNA) by 79.4±3.9% (Fig. 5A), which on the protein level amounted to a 72.5±3.5% decreased expression, without significantly affecting Ca_V_1.2 expression (Fig. 5B and C). However, the most striking finding was that silencing of eIF3e almost fully prevented glucose-stimulated internalization of Ca_V_1.2 clusters and the PM/C ratio remained unchanged upon exposure to 20 mM glucose when explored by immunocytochemistry (Fig. 5D and E). To better visualize the dynamics of Ca_V_1.2 in the PM we next performed experiments using total internal reflection microscopy (TIRFM; Fig. 5F, G and H). This imaging technique selectively visualizes fluorescent molecules within ∼100 nm distance from the PM. First, these experiments showed that eIF3e silencing resulted in a slight, but non-significant, reduction in Ca_V_1.2 cluster number at the PM. Second they confirmed the capacity of glucose stimulation to reduce Ca_V_1.2 cluster number at the PM. Third, they demonstrated that glucose-induced internalization of Ca_V_1.2 clusters at the PM effect was prevented by silencing of eIF3e. Interestingly, the whole-cell Ca^2+^ current-voltage relations (I-Vs) in Figure 5I show the failure of long-term glucose treatment to reduce voltage-stimulated Ca^2+^ influx in eIF3e KD cells. These results underscore the physiological role of eIF3e to control surface expression of Ca_V_ channels such as Ca_V_1.2.

### eIF3e Assists Cav1.2 Trafficking to the Plasma Membrane

**Figure 6:**
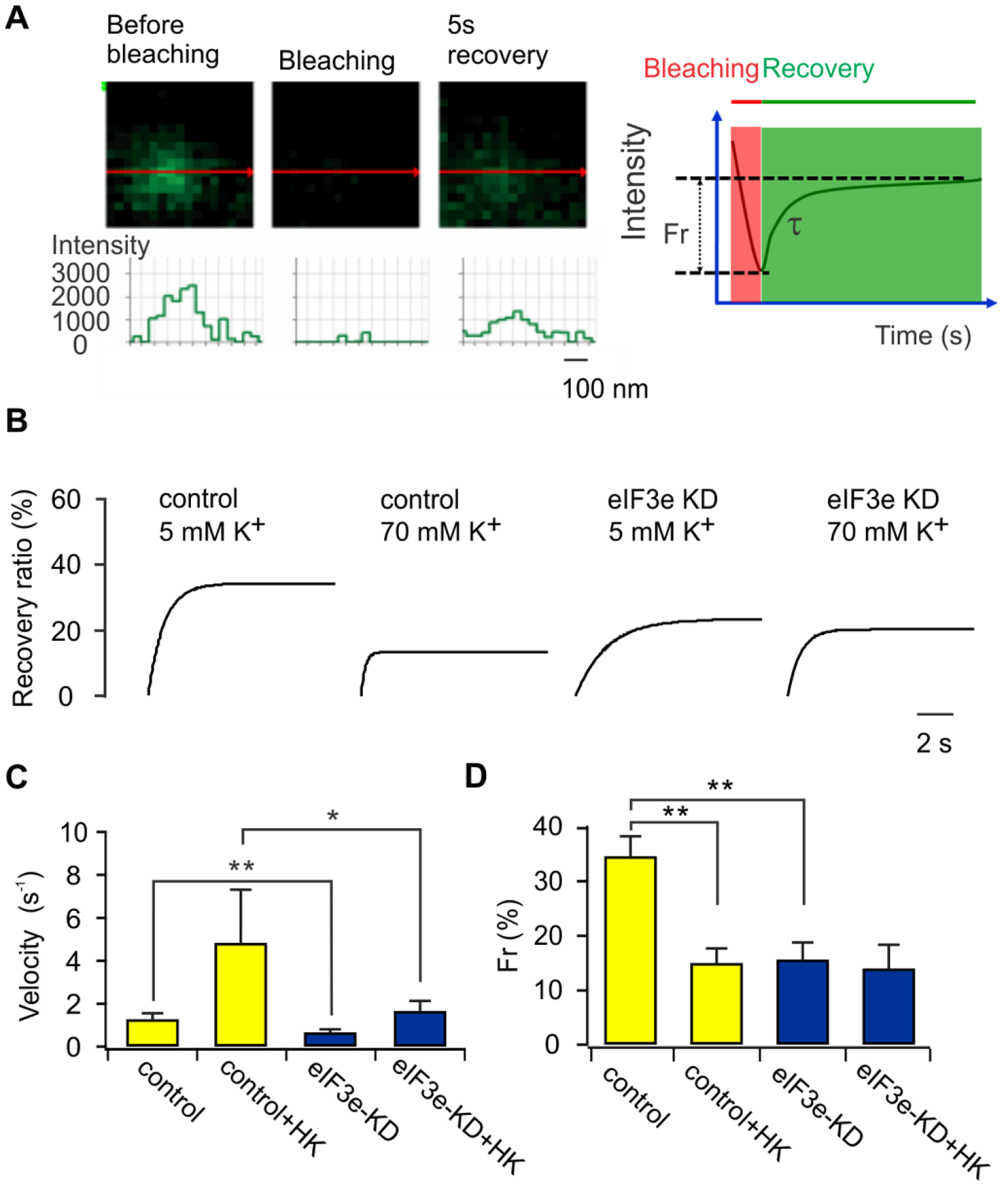
eIF3e silencing reduces Cav1.2 cluster transport to the plasma membrane. A) Arrival of a single Ca_V_1.2 cluster to the PM revealed by confocal imaging after FRAP. The three images show (left to right) cluster fluorescence before, immediately after photobleaching, as well as after 5-s recovery. The curves below denote the fluorescence intensity along the red lines in the confocal images. Schematic representation (right) of amount of recovery Ca_V_1.2 molecules (Fr) and recovery time constant (τ) in a single FRAP event. B) FRAP in control and eIF3e KD cells at 5 and 70 mM K^+^. Depolarization by 70 mM K^+^ increases the velocity of Ca_V_1.2 cluster transport to the PM, when expressed as the rate of recovery after photobleaching. The curves denote the best exponential fit to average fluorescence intensity recovery trends. C) Average velocity (1/τ) of the recovery in 27 events for each experimental group. D) Average recovery of fluorescence intensity of Ca_V_1.2 molecules (Fr) in 27 events for each experimental group. All data are expressed as averages± S.E.M. * p<0.05, ** p<0.01.(ANOVA, F-test).

Since the number of Ca_V_1.2 clusters are affected by bi-directly intracellular trafficking of Ca_V_1.2 we therefore investigated whether eIF3e is involved in the regulation of Ca_V_1.2 trafficking to PM. To detect movement of Ca_V_1.2 clusters to the PM, we designed a modified Fluorescence Recovery After Photobleaching (FRAP) protocol to track single cluster movements (Fig. 6A). After photobleaching, two possibilities for Ca_V_1.2 fluorescence recovery in the bleached area exist: first, the possibility of lateral diffusion in the X–Y plane, implies that fluorescence recovery should first appear along the edges. The other option is that cluster replenishment occurs from the cytosol along the Z-axis, which means that the fluorescence increase should appear in a central spot. We obtained strong evidence for cluster recovery both by lateral diffusion, as well as from the cell interior (Fig. 6A). The latter type of events was selected to further study fluorescence recovery over time, which was fitted to an exponential function (Fig. 6B). This analysis clearly showed that the fluorescence recovery was faster after stimulation with 70 mM K^+^ for 30 min, as demonstrated by the 4-fold increased velocity of recovery when compared to that observed at normal K^+^ concentration (from 1.25±0.29 s^−1^ to 4.82±2.5 s^−1^; n = 27 events under each condition) (Fig. 6C). However, it is important to note that the amount of recovery was only half of that observed in unstimulated control cells (Fig. 6D). This suggests that depolarization increases the velocity of Ca_V_1.2 recovery to the PM but also limits the amount of Ca_V_1.2 clusters available for replenishment (recovery fluorescence intensity decreased from 35.5±0.09% to 14.9±0.07%; n = 27 events under each condition). Silencing of eIF3e slowed Ca_V_1.2 trafficking and the velocity of recovery decreased under both resting (0.64±0.16 s^−1^; n = 27) and activated conditions (1.60±0.53 s^−1^; n = 27), respectively (Fig. 6C). The finding that eIF3e silencing decreases the speed and absolute amount of Ca_V_1.2 trafficking to the PM, implies that eIF3e is a factor that assists Ca_V_1.2 transport, whilst also controlling the size of the pool of Ca_V_1.2 channel clusters available for recovery.

### eIF3e Prevents Ca^2+^ Overload

**Figure 7:**
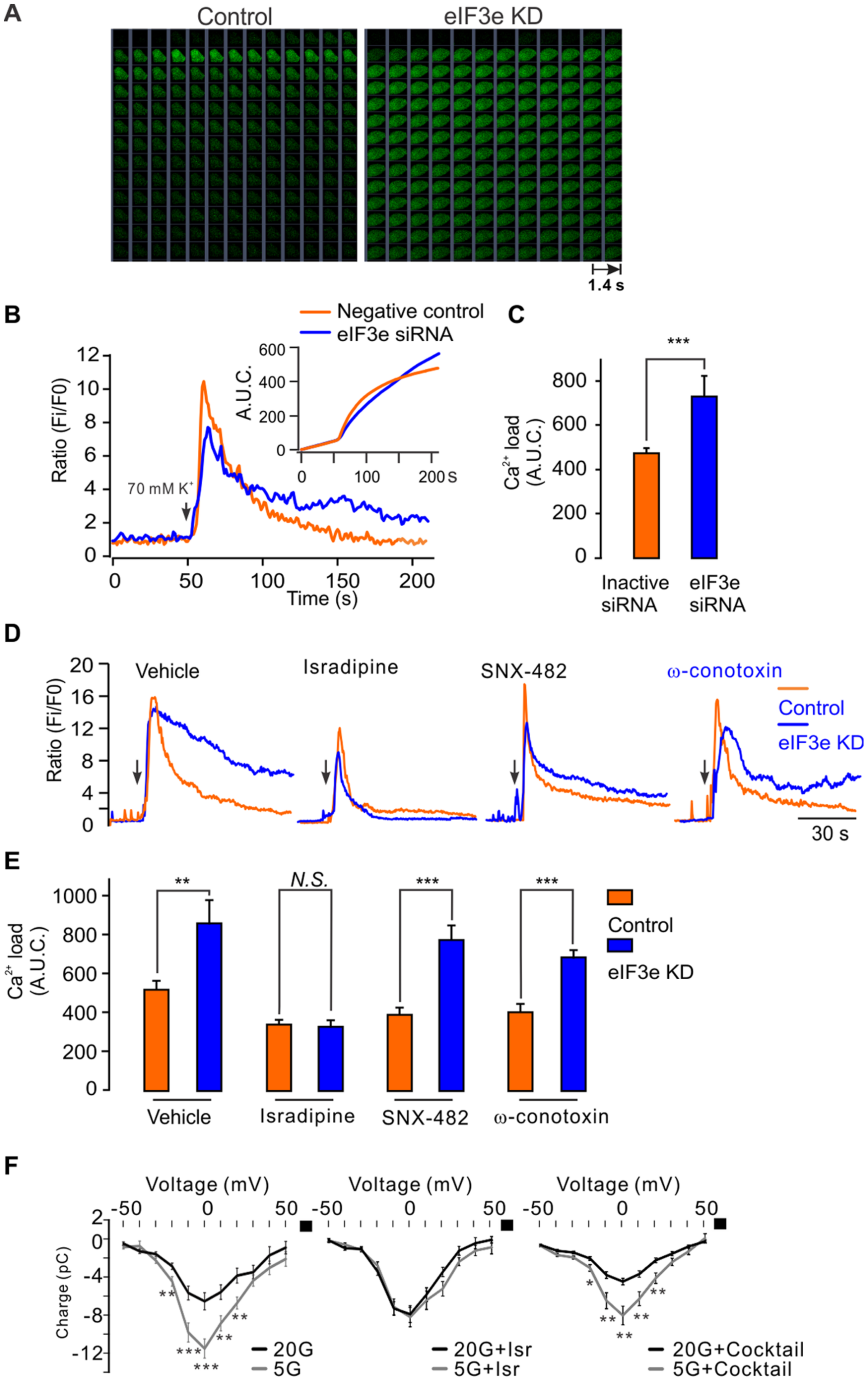
eIF3e silencing disrupts intracellular Ca^2+^ homeostasis. A) Images of intracellular Ca^2+^ measured by the low affinity calcium dye, Fluo-5F upon 70 mM K^+^ stimulation in control and eIF3e KD cells. B) Fluo-5F fluorescence signal ratio (Ratio) and its time integral (A.U.C.) were calculated to assess the Ca^2+^ influx. Note that after 180-s stimulation the integrated Ca^2+^ load in eIF3e KD cells exceeds that of the negative control-treated cells. The arrow indicates the onset of stimulation. C) Comparison of Ca^2+^ load, expressed as the Area Under the Curve (A.U.C) 0-200 s after stimulation, between eIF3e KD and control cells (n = 27 cells, ***, P<0.001; Student’s t-test). D) As in (a), but experiments performed in the presence of vehicle (DMSO), the L-type calcium channel inhibitor isradipine (5 µM), the R-type calcium channel inhibitor SNX482 (0.2 µM) or the N-type calcium channel inhibitor ω-conotoxin GVIA (50 nM). Note that isradipine counteracts the effects of eIF3e KD on the sustained rise in cytosolic [Ca^2+^]. E) Quantitative analysis of Ca^2+^ load as in C for the conditions in D; (n = 18 cells for each condition, **, P<0.01; ***, P<0.001; Student’s t-test). F) Current-voltage (I-V) relations for whole-cell Ca^2+^ currents after 30 min incubation in 5 or 20 mM glucose (left); same experiment but in the presence of 5 µM isradipine (middle); or a non-L-type blocker cocktail (0.1 µM ω-agatoxin, 0.2 µM SNX-482 and 50 nM ω-conotoxin GVIA; right). n = 11 or more for each condition. * P<0.05, ** P<0.01, *** P<0.001 (Student’s t-test).

The finding that Ca_V_1.2 clusters in eIF3e-silenced cells are not internalized suggests that this condition could potentially be associated with Ca^2+^ overload during long-term stimulation. To explore this possibility, we used the low-affinity Ca^2+^ dye Fluo-5F for confocal Ca^2+^ imaging in both control and eIF3e-silenced cells (Fig. 7A). Upon stimulation with 70 mM K^+^ an initial peak in intracellular Ca^2+^ ([Ca^2+^]_i_) was followed by a rapid decline reaching a plateau after ∼50 s (Fig. 7B). The initial [Ca^2+^]_i_ peak was higher in control cells treated with inactive siRNA, but from ∼3 min after onset of the stimulation, the time integral of the Ca^2+^ signal in eIF3e knock-down cells exceeded that observed in control cells (Fig. 7B; inset). These results demonstrate that during long-term stimulation, the total Ca^2+^ influx in eIF3e knock-down cells is significantly elevated compared to control-treated cells (729±93 vs 474±46 AU*s, 27 cells in each group) (Fig. 7C). To determine whether the increase of Ca^2+^ influx specifically reflects the localization of L-type channels, including Ca_V_1.2, we used pharmacological channel inhibitors to detect the Ca_V_ subtype-dependence of the effect of eIF3e silencing (Fig. 7D and E). In cells stimulated by high K^+^, eIF3e siRNA caused long-term Ca^2+^ influx and significantly raised the Ca^2+^ load (from 517±45 to 855±121 AU*s, 18 cells in each group). Interestingly, the L-type calcium channel blocker isradipine counteracted the increase of Ca^2+^ load in eIF3e-silenced cells (342±19 vs 333±32 AU*s; n = 18 in each group). However, neither the R-type calcium channel blocker SNX-482, or the N-type calcium channel blocker w-cototoxin GVIA were able to prevent the increased Ca^2+^ load caused by eIF3e silencing (Fig. 7D and E). We also measured whole-cell Ca^2+^ current-voltage relations (I-Vs) to investigate the effects of the blockers on the capacity of long-term glucose treatment to suppress Ca^2+^ influx (Fig. 7F, left). We first observed that the L-type blocker isradipine overall decreased whole-cell Ca^2+^ currents by ∼50%. Interestingly, long-term glucose treatment now failed to further reduce Ca^2+^ influx (Fig. 7F, middle). Secondly, a cocktail of non-L type Ca_V_ channel blockers (R-channel blocker SNX-482, P/Q-channel blocker ω-agatoxin IVA and N-channel blocker ω-cototoxin GVIA) reduced whole-cell Ca^2+^ currents to a similar extent as isradipine. Important to note is the preserved capacity of glucose treatment to further reduce Ca^2+^ influx under these conditions (Fig. 7F, right). These results support the view that in insulin-secreting cells L-type Ca_V_ channels, e.g. Ca_V_1.2, form the most important Ca_V_ channel population in activity-dependent internalization.

## Discussion

### Regulation of Ca_V_1.2 Cluster Surface Expression

The present data suggest that L-type Ca_V_ channel internalization, as exemplified by Ca_V_1.2, is a physiological phenomenon, and one that is used as a mode of negative feedback upon stimulation by e.g. glucose. Ca_V_1.2 cluster surface expression was significantly lowered after glucose stimulation for 15 min or longer (Figs. 1 and 2). These results add another facet to the physiological function of glucose in β-cells. We wish to emphasize that although this study details the mechanisms whereby Ca_V_1.2 is internalized under stimulatory conditions, we wish not to exclude the participation of other L-type Ca_V_ channels in the same process. For example, Ca_V_1.3 is likewise highly expressed in insulin-secreting cells (although functionality appears to be species-dependent) and may very well contribute to the increased intracellular Ca^2+^ load observed after interfering with Ca_V_ trafficking. With present L-type blockers it is not possible to discriminate between different L-type channel species. Moreover, in neurons, also non-L-type Ca_V_ channels appear to be subject to stimulation-dependent internalization [18]. However, in insulin-secreting cells this effect is difficult to discern, which perhaps is a consequence of their lower relative expression. Be that as it may, the ability to internalize specific Ca_V_ channel subtypes offers a dynamic and rapid mode of regulating the physiology of the β-cell and could, for example, explain the shift from L- to R-type Ca_V_ channel dependence during phasic insulin secretion in mouse [8].

The activity-dependent regulation of Ca_V_ channel surface expression appears to be mediated by Ca^2+^-dependent signals, since stimulation with high K^+^ has effects similar to glucose. However, many details remain unresolved, such as the fate of the internalized clusters. In general, two main possibilities exist, the first being that Ca_V_1.2 is dispersed into single molecules and degraded by ubiquitin-dependent proteases through endoplasmic reticulum–associated protein degradation [25]. It is worthy of note that our unpublished experiments using the protease inhibitor Mg132 suggest that this is not the fate of the majority of internalized Ca_V_1.2 clusters. The other possibility is that Ca_V_1.2 clusters are internalized by the clathrin-dependent endocytotic pathway, which is expected to recycle the Ca_V_1.2 channels via this standby pool of inactive Ca_V_1.2 channels, back to their active state in the PM in response to environmental cues [26]. This alternative is supported by the observed interaction with clathrin and the early endosome marker EEA-1 (Fig. 1D and 3A), as well as the experiments using inhibitors of microtubules or dynamin (Fig. 3E and F). Taken together with the recovery of Cav1.2 expression in the PM (Fig. 2) after wash-out, these results collectively suggest that Ca_V_1.2 is subject to dynamic regulation of its expression in the PM.

### Ca_V_1.2 Trafficking, Ca^2+^ Homeostasis and β-cell Function

Like the Ca_V_1.2 clusters, eIF3e internalizes in an activity-dependent fashion. The physical interaction of eIF3e and Ca_V_1.2 is evident from immunoprecipitation experiments (Fig. 4E), and probably occurs via a binding domain in the II-III loop of the Ca_V_1.2 alpha subunit [18]. Furthermore, silencing of eIF3e prevents glucose-dependent Ca_V_1.2 cluster internalization (Fig. 5D). These results collectively provide strong evidence for the physical and functional connection between Ca_V_1.2 and eIF3e.

eIF3e is a regulatory subunit in the protein translation initiation complex. Therefore its silencing could be envisioned to affect Ca_V_1.2 subcellular expression merely as a secondary effect to this fundamental function. However, it should be noted that eIF3e is not a required subunit in the complex and its silencing will not prevent protein synthesis [15], [24]. Accordingly, silencing of eIF3e only resulted in a relatively modest reduction of the number of Ca_V_1.2 clusters. Furthermore, the fact that insulin secretion was largely unaffected in eIF3e-silenced cells (data not shown) adds further impetus to the idea of a specific regulatory action exerted by eIF3e. Finally, activity-dependent Ca_V_1.2 internalization (and its reversal) was totally unaffected in cells treated with the protein synthesis inhibitor cycloheximide (Fig. 4D). These data taken together strongly support the idea that eIF3e serves as a physiological modulator in the β-cell, controlling vesicular transport of Ca_V_1.2 clusters, and possibly other L-type Ca_V_ channels, to the PM and their internalization under periods of intense stimulation. Glucose-dependent insulin secretion involves activation of Ca_V_ channels and requires substantial increases in local [Ca^2+^]_i_ in the nanodomains in which Ca_V_ channels and insulin granules aggregate prior to release [27]. However, during protracted stimulation spillover from the nanodomains could lead to alarmingly high global cellular [Ca^2+^]_i_ that could trigger excitotoxic effects and eventually apoptosis [5]. Therefore a mechanism whereby Ca_V_ channel surface expression can be adjusted during intense activity offers an important cellular cut-off reaction for limiting Ca^2+^ influx. In conclusion, the ability of the β-cell to regulate cell surface expression of L-type channels such as Ca_V_1.2 will have consequences for β-cell Ca^2+^ homeostasis and further work will determine its relevance for progression of type 2-diabetes.

## Materials and Methods

### Cell Culture and Transfection

Clonal INS-1 832/13 cells were cultured as previously described [28]. 0.5 µg N-terminally EGFP-tagged Ca_V_1.2 and Ca_V_2.1 [29], [30] were transiently transfected using 1.5 µl Lipofectamine 2000 (Invitrogen, CA, USA) per well in 24-well plate. After 24 h transfection, the cells were re-seeded on a 0.175 µm thickness glass and followed up 12 h culture until images acquirement.

### RNA Interference

INS-1 832/13 cells were seeded 1 day prior to transfection. 30 nM eIF3e RNA interference oligonucleotides (Applied Biosystems) or 30 nM control #1 (Applied Biosystems, USA) were used to silence eIF3e. The siRNA was transfected by using Dhamafect Kit (Thermo Scientific, USA). The transfection efficiency was estimated by BLOCK-It AlexaFluorRed (Invitrogen, CA, USA) which stains the transfected cells. After 48 h transfection, the cells were collected and total RNA was extracted by using a RNA extraction kit (Qiagen, Germany). 1 µg RNA was used for RT-PCR and real time PCR. Primers of eIF3e and housekeeping gene HPRT1 (Applied Biosystems, USA) which tagged FAM dyes were used for amplification detection.

### Immunostaining

Cells were first washed twice and fixed with 3% PFA-PIPES and 3% PFA-Na_2_BO_4_ for 5 min and 10 min respectively, followed by permeabilization with 0.1% Triton-X 100 for 30 min. The blocking solution contained 5% normal donkey serum in PBS and was used for 15 min. Primary antibodies against Ca_V_1.2 (Sigma, USA), EEA-1 (BD transduction lab, USA), Na^+^/K^+^ ATPase (Millipore, USA), Rab11 (BD transduction lab, USA), clathrin (BD transduction lab, USA), Dynamin (Millipore, USA), Tubulin (Sigma, USA), eIF3e (Santa Cruz, CA) were diluted in blocking solution and incubated overnight at 4°C. Immunoreactivity was done using fluorescently labeled secondary antibodies (1∶200) and visualized by confocal microscopy (Carl Zeiss, Germany). Colocalization analysis was performed by using a ZEN2009 software based on Pearson’s correlation coefficient analysis which recognizes the colocalized pair by comparison pixel by pixel intensity [31]. The internalization was indicated by ratio that is defined by mean intensity of plasma membrane to mean intensity in cytosol, according to the formula: Ratio = 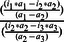. Where *i_1_, i_2_* and *i_3_* represent the intensities of whole cell, cytosol and nucleus, *a_1_*_,_ *a_2_* and *a_3_* represent the area of whole cell, cytosol and nucleus respectively. The specificities of Ca_V_1.2 and eIF3e antibodies were validated by using synthesized peptides which totally blocked the signals. Others antibody stainings were performed following protocols provided by the vendor.

### Electrophysiology

Whole-cell Ca^2+^ currents were measured as described previously [28]. The extracellular solution (118 mM NaCl, 20 mM tetraethylammonium chloride, 5.6 mM KCl, 2.6 mM CaCl_2_, 1.2 mM MgCl_2_ and 5 mM HEPES) was supplemented with 5 or 20 mM glucose as indicated. The pipette (intracellular) solution contained 125 mM Cs-glutamate, 10 mM CsCl, 10 mM NaCl, 1 mM MgCl_2_, 5 mM HEPES, 3 mM Mg-ATP, 0.1 mM cAMP and 0.05 mM EGTA (pH 7.2 with CsOH). L-type blocker isradipine (5 µM), R-channel blocker SNX-482 (200 nM), P/Q-channel blocker ω-agatoxin IVA (100 nM) and N-channel blocker ω-cototoxin GVIA (50 nM) were added as indicated in text or figures. Data was recorded on a HEKA EPC9 patch clamp amplifier with the Pulse Fit 8.64 software. The whole-cell configuration was used in voltage-clamp mode and pipettes had an average resistance of ≈5.5 MΩ. All the experiments were performed in bath-heated perfusion system which controls output temperature to 32°C.

### Subcellular Fractionation

Subcellular fractionation of INS-1 832/13 cells was done as described previously [32]. Briefly, cells were scraped into 15 ml ice cold homogenization medium (HM; 250 mM sucrose, 5 mM HEPES, 0.5 mM EGTA, 0.2 mM PEFA Block and adjusted to pH 7.4 with KOH) and disrupted using a nitrogen bomb (350 psi). The homogenate was centrifuged at 700×g for 15 min in 4°C and postnuclear supernatant was separated. 15% (v/v) Percoll and 250 mM sucrose were added into the mix and super-centrifuged at 48,000×g for 25 min at 4°C. Two opaque bands were obtained at the top and bottom corresponding to the plasma membrane and vesicles, respectively. The fractions were sonicated and total protein concentration was measured using BCA protein assay kit (Pierce, IL, USA).

For immunoblotting, 40 µg of the total proteins was loaded onto 7.5% SDS-PAGE gels. Blotting was carried out by incubation overnight at 4°C with polyclonal anti-Ca_V_1.2 antibody (1∶500), anti-clathrinhevay chain (Abcam, 1∶200), caveolin-1 (SantaCruz, 1∶200) followed by incubation with horseradish peroxidase-conjugated secondary antibodies (1∶5,000; Pierce) or Clean Blot 21230 (ThermoScientific, 1∶200) for at least 1 hour. Final signal was indicated by SuperSignal West Pico Chemiluminescent Substrate (Pierce, IL, USA).

For immunoprecipitation (IP), INS-1 832/13 cells were lysed in 500 µl of the lysis buffer (0.2% Triton-X 100, 0.2% NP-40, 150 mM NaCl, 1 mM EGTA, 50 mM Tris pH 7,5, cocktail of protease inhibitors (Roche, Basel, Switzerland)). 25 µg obtained protein solution was incubated with agitation overnight at 4°C with anti-Ca_V_1.2antibodies covalently coupled with 5 mg Dynabeads (Invitrogen, CA, USA). The eluted fraction was immunoblotted as described above.

### Live Cell Imaging

Cells seeded onto cover slips and mounted in the experimental chamber were perifused and temperature-controlled during the entire experiment. Confocal images were acquired using a Zeiss 510 Meta LSM and a ×40 water immersion objective (NA = 1.2). EGFP-Ca_V_1.2 was visualized by excitation at 488 nm and collected using a 500–530 nm bandpass filter. The pinhole was ∼1 airy unit and the scanning frame was 512×512 pixels. Colocalization analysis was evaluated by Pearson’s correlation coefficient analysis using the ZEN 2009 software (Zeiss, Germany). For z-stack acquisition, we used a low power laser output (<2%) and stack interval at 0.5 µm. The middle sections in a cell were selected for ratio (PM/C) analysis in which the area of the plasma membrane (PM) was defined using the PM marker CellMask (Invitrogen, USA). 3D reconstruction was performed by ZEN2009. For Fluorescence Recovery After Photobleaching (FRAP) experiments, we first adjusted the focus on the cell-cover slip interface and carefully shifted focus to the cell surface. Image acquisition in EGFP-Ca_V_1.2 transfected cells was done using a minimum pinhole (section thickness 0.6 µm) to observe single Ca_V_1.2 clusters in Ca_V_1.2 overexpressing cells. Next, photobleaching performed by full argon laser outputs, as determined in the region of interests (ROIs) in the center of the visual fields. Finally the recovered fluorescent intensity was analyzed using ZEM 2009 and exponential fitting was performed using the Igor software (Portland, USA).

### Ca^2+^ Imaging

Fluo-5F (K_d_ = 2.3 µM) (Invitrogen, USA) was used for measuring intracellular Ca^2+^. Cells were loaded using 1 µM Fluo-5F in room temperature for 30 min. The cells first were perfused in Krebs-Ringer bicarbonate (KRB) buffer containing 116 mM NaCl, 4.7 mM KCl, 2.6 mM CaCl_2_, 1.2 mM KH_2_PO_4_, 1.2 mM MgSO_4_, 20 mM NaHCO_3_, 16 mM HEPES, 2 mg/ml BSA, and supplemented with 5 mM glucose. Stimulation was carried out by a 70 mM KCl KRB buffer at room temperature. Images were acquired by confocal microscopy using a 40× water immersion objective. A ratio was calculated by taking the fluorescence intensity in time lapse divided by the average fluorescence intensity under pre-stimulatory conditions. The time integral of the fluorescence signal (Area Under the Curve, A.U.C) was calculated using formula: 
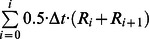
, where 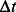, the time interval; 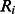, the ratio at time point *i*.

### Total Internal Reflection Fluorescencemicroscopy (TIRFM) Imaging

INS-1 cells were seeded on glass bottom MatTek dishes, and stainined for Ca_V_1.2 by the normal immunocytochemistry protocol detailed above. TIRFM images were acquired by a high-aperture 100× objective lens in an inverted epifluorescence microscope (Carl Zeiss). Before image acquisition, the penetration depth of the evanescent wave was determined by 3 µm fluorescent beads. To visualize the Cy 2-labeled Ca_V_1.2, the cells were excited using the 488-nm line of an argon laser and a 515 nm long pass emission filter. The images were collected by a CCD camera (COOLSNAP, Photometrics, UK) operated by the MetaMorph software (Molecular device, USA). Image analysis was done using ImageJ freeware (NIH, USA) and the number of Ca_V_1.2 clusters were calculated by using the cell counter plug-in.

### Statistical Analysis

The data were shown present as averages±S.E.M. Evaluation of statistical significance was done by Student’s T-test for experiments allowing paired comparisons, or by one-way analysis of variance (ANOVA) with the Friedman test for multiple comparisons.

## Supporting Information

Figure S1 **Detection of Cav1.2 surface expression by total internal reflection fluorescence microscopy (TIRFM).** A) Representative TIRFM image of Ca_V_1.2 in control and after 30-min stimulation with 20 mM glucose or 70 mM KCl. B) Representative TIRFM image of Na^+^/K^+^ ATPase under the conditions as in A. C) Quantitative analysis of Ca_V_1.2 clusters in control or stimulated conditions. Data indicate the number of Ca_V_1.2 clusters per cell and are presented as averages±S.E.M. D) Quantitative analysis of Na^+^/K^+^ ATPase clusters under conditions as in C. n = 17, *** p<0.001 (ANOVA F-test).
(TIF)

Figure S2 (TIF)**Visualization of Cav1.2 surface expression in EGFP-Cav1.2 transfected INS-1 cells by TIRFM imaging.** A) Representative TIRFM image series of Ca_V_1.2 in the conditions with stimulation of vehicle, 20 mM glucose or 70 mM KCl. The histograms show the number of Cav1.2 clusters per cell under control conditions B, in cells exposed to 70 mM KCl (C) or 20 mM glucose (D). n = 11, ** p<0.01, *** p<0.001 (ANOVA, F-test).
(TIF)

Figure S3 **Surface expression of eIF3e revealed by TIRFM imaging.** A) Representative image of eIF3e under control conditions and stimulation by vehicle, 20 mM glucose or 70 mM KCl. B) Quantitative analysis of eIF3e cluster number on the INS-1 cell surface. Data indicate the number of eIF3e clusters per cell and are presented as averages±S.E.M. n = 17, *** p<0.001 (ANOVA F-test).
(TIF)
