# Supplementary figures and images for: Eukaryotic Translation Initiation Factor 3 Subunit E Controls Intracellular Calcium Homeostasis by Regulation of Cav1.2 Surface Expression
Source: PLoS One. 2013 May 30;8(5):e64462. doi: 10.1371/journal.pone.0064462 (PMC3667822; doi:10.1371/journal.pone.0064462)

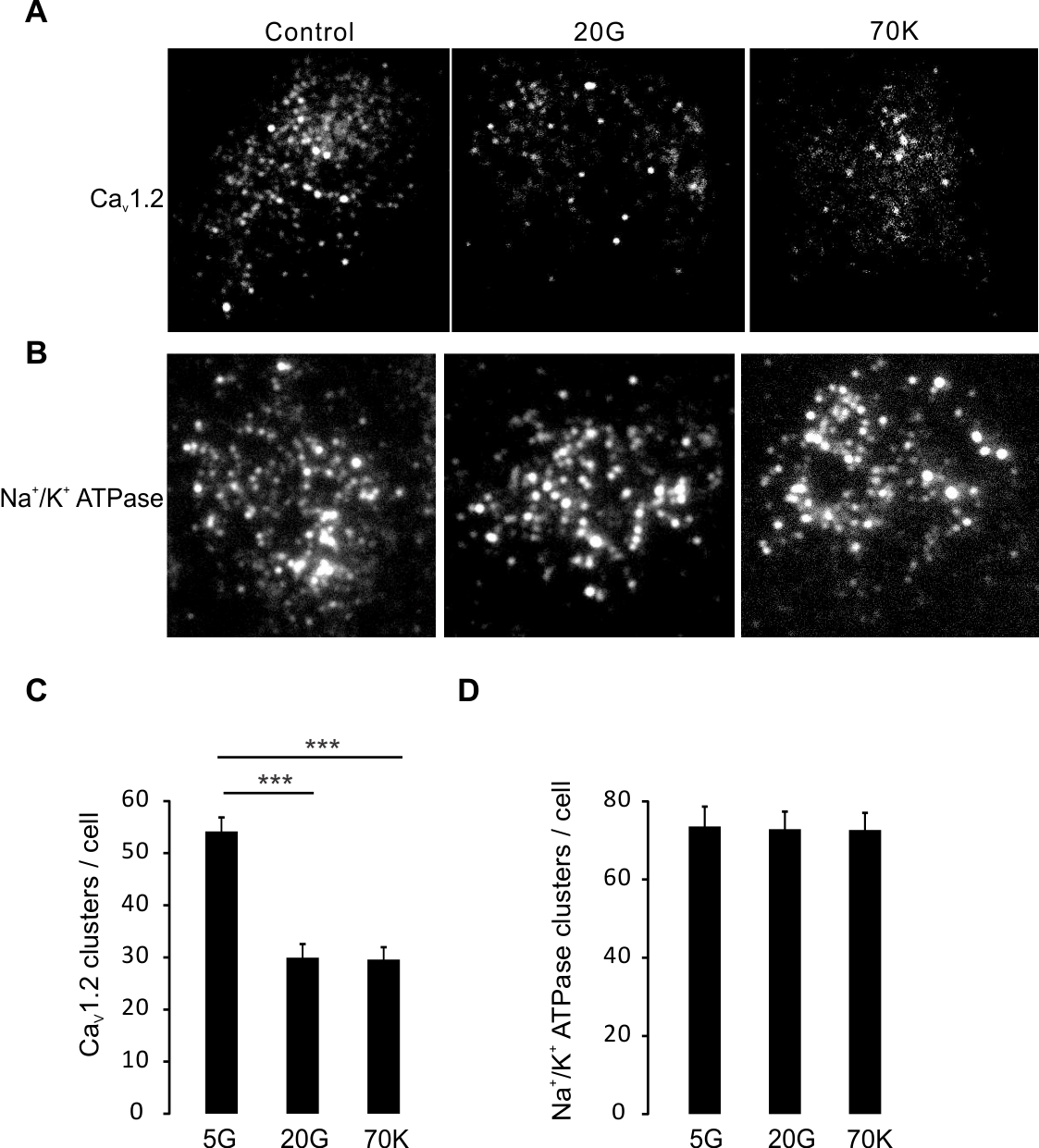

Supplement: Figure S1 — Detection of Cav1.2 surface expression by total internal reflection fluorescence microscopy (TIRFM). A) Representative TIRFM image of CaV1.2 in control and after 30-min stimulation with 20 mM glucose or 70 mM KCl. B) Representative TIRFM image of Na+/K+ ATPase under the conditions as in A. C) Quantitative analysis of CaV1.2 clusters in control or stimulated conditions. Data indicate the number of CaV1.2 clusters per cell and are presented as averages±S.E.M. D) Quantitative analysis of Na+/K+ ATPase clusters under conditions as in C. n = 17, *** p<0.001 (ANOVA F-test). (TIF) [file pone.0064462.s001.tif]

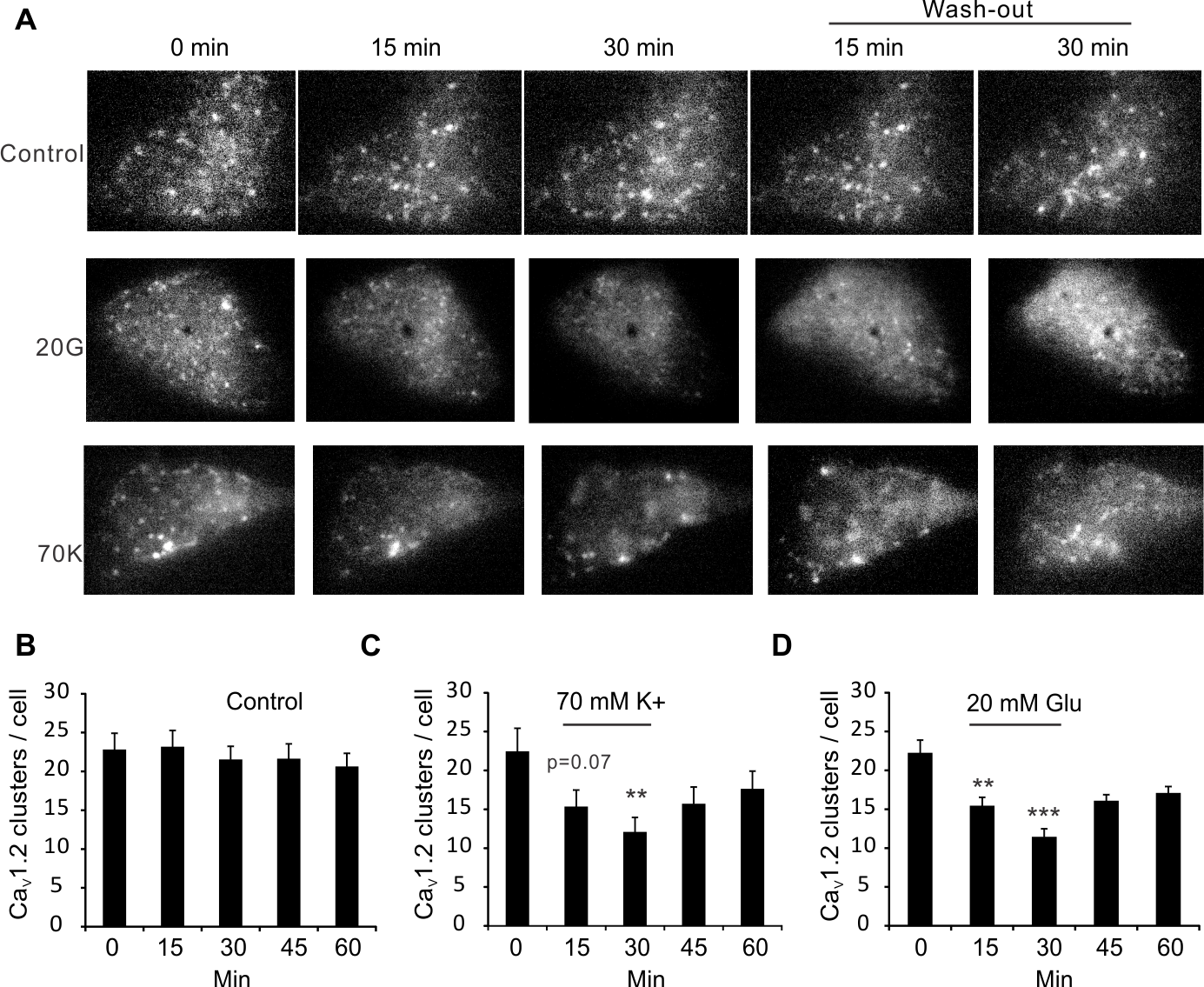

Supplement: Figure S2 — (TIF)Visualization of Cav1.2 surface expression in EGFP-Cav1.2 transfected INS-1 cells by TIRFM imaging. A) Representative TIRFM image series of CaV1.2 in the conditions with stimulation of vehicle, 20 mM glucose or 70 mM KCl. The histograms show the number of Cav1.2 clusters per cell under control conditions B, in cells exposed to 70 mM KCl (C) or 20 mM glucose (D). n = 11, ** p<0.01, *** p<0.001 (ANOVA, F-test). (TIF) [file pone.0064462.s002.tif]

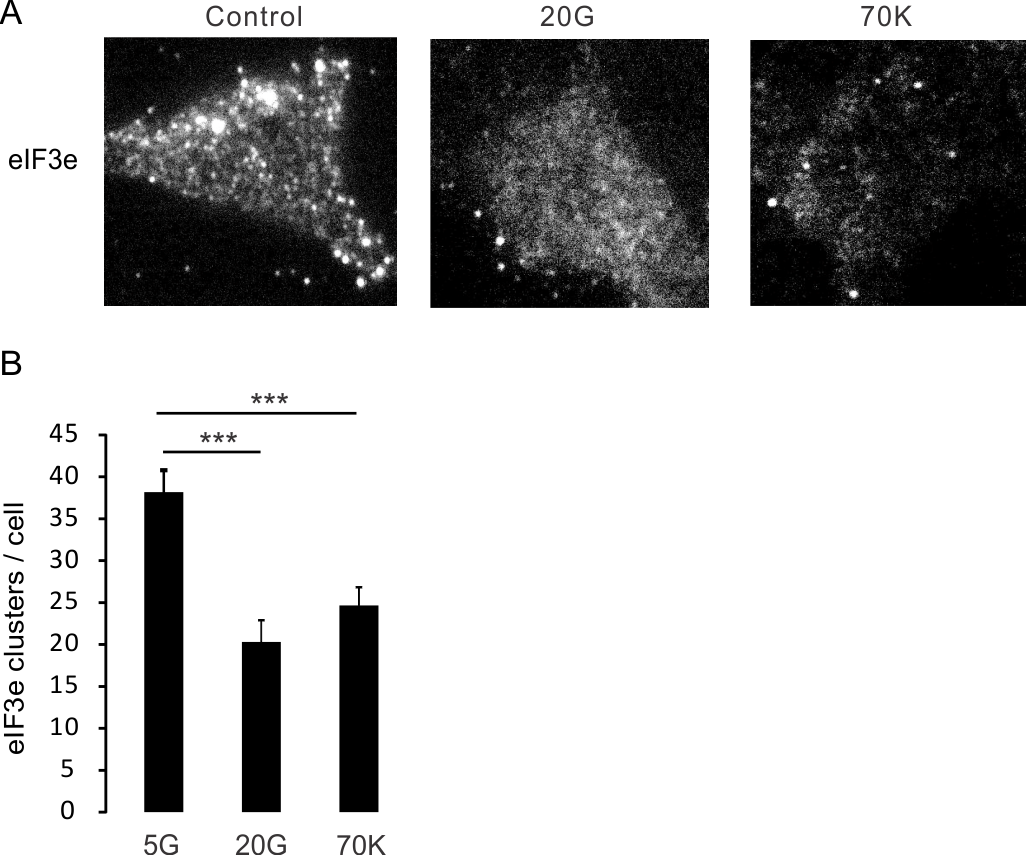

Supplement: Figure S3 — Surface expression of eIF3e revealed by TIRFM imaging. A) Representative image of eIF3e under control conditions and stimulation by vehicle, 20 mM glucose or 70 mM KCl. B) Quantitative analysis of eIF3e cluster number on the INS-1 cell surface. Data indicate the number of eIF3e clusters per cell and are presented as averages±S.E.M. n = 17, *** p<0.001 (ANOVA F-test). (TIF) [file pone.0064462.s003.tif]
